# Corrigendum: An extra-uterine system to physiologically support the extreme premature lamb

**DOI:** 10.1038/ncomms15794

**Published:** 2017-05-23

**Authors:** Emily A. Partridge, Marcus G. Davey, Matthew A. Hornick, Patrick E. McGovern, Ali Y. Mejaddam, Jesse D. Vrecenak, Carmen Mesas-Burgos, Aliza Olive, Robert C. Caskey, Theodore R. Weiland, Jiancheng Han, Alexander J. Schupper, James T. Connelly, Kevin C. Dysart, Jack Rychik, Holly L. Hedrick, William H. Peranteau, Alan W. Flake

Nature Communications **8**: Article number: 15112; DOI: 10.1038/ncomms15112 (2017); Published 04 25 2017; Updated 05 23 2017

A patent based on the work reported in this Article was inadvertently omitted from the Competing interests section of this article. The Competing interests statement should read:

E.M., A.F. and M.D. are co-authors on a patent entitled ‘Extracorporeal life support system and methods of use thereof' (Patent no. WO2014145494 A1). The remaining authors declare no competing financial interests.

